# Diversified Floral Resource Plantings Support Bee Communities after Apple Bloom in Commercial Orchards

**DOI:** 10.1038/s41598-019-52601-y

**Published:** 2019-11-21

**Authors:** Sarah Heller, Neelendra K. Joshi, Timothy Leslie, Edwin G. Rajotte, David J. Biddinger

**Affiliations:** 1 Fruit Research & Extension Center, Entomology, Pennsylvania State University, 290 University Dr, Biglerville, 17307 PA USA; 2 Department of Entomology, 501 ASI Building, Pennsylvania State University, University Park, 16802 PA USA; 3 Present Address: Department of Entomology and Plant Pathology, 217 Plant Sciences Building, University of Arkansas, Fayetteville, Arkansas 72701 USA; 4 Department of Biology, Long Island University, 1 University Plaza, Brooklyn, New York, 11201 USA; 5 Present Address: USDA APHIS PPQ Otis Laboratory, 1398 West Truck Road, Buzzards Bay, Massachusetts 02542 USA

**Keywords:** Agroecology, Entomology, Biodiversity

## Abstract

Natural habitats, comprised of various flowering plant species, provide food and nesting resources for pollinator species and other beneficial arthropods. Loss of such habitats in agricultural regions and in other human-modified landscapes could be a factor in recent bee declines. Artificially established floral plantings may offset these losses. A multi-year, season-long field study was conducted to examine how wildflower plantings near commercial apple orchards influenced bee communities. We examined bee abundance, species richness, diversity, and species assemblages in both the floral plantings and adjoining apple orchards. We also examined bee community subsets, such as known tree fruit pollinators, rare pollinator species, and bees collected during apple bloom. During this study, a total of 138 species of bees  were collected, which included 100 species in the floral plantings and 116 species in the apple orchards. Abundance of rare bee species was not significantly different between apple orchards and the floral plantings. During apple bloom, the known tree fruit pollinators were more frequently captured in the orchards than the floral plantings. However, after apple bloom, the abundance of known tree fruit pollinating bees increased significantly in the floral plantings, indicating potential for floral plantings to provide additional food and nesting resources when apple flowers are not available.

## Introduction

Insect pollinators are essential in nearly all terrestrial ecosystems, and the ecosystem services they provide are vital to both wild plant communities and agricultural crop production. Bees are the primary insect pollinators in agricultural ecosystems, and they provide an estimated global service to food production worth $215 billion^1^. Most crop producers have relied on managed bees, such as the honey bee (*Apis mellifera* L.) for many years and in most situations, but reliance on this single species for commercial pollination is threatened by high rates of colony loss over the last decade^2,3^. Concurrent declines of wild bee populations, although more difficult to measure, also raise concerns regarding pollination deficits^4–6^. Bee declines are most likely caused by the exposure to multiple interacting stressors such as pesticide use, pathogens, parasites, and a reduction in appropriate floral and nesting resources^7^.

Many factors may limit access to the feeding and nesting resources that bees need^7^. Loss of the habitat providing these resources has been a long-term contributor to bee declines^8^. Many bee species may respond favorably to moderate landscape disturbances that create nesting sites and stimulate floral diversity^9^. Extreme urbanization and monoculture cropping create a fragmented habitat and limited diet breadth both nutritionally and temporally, which can be generally unfavorable to managed and wild bees^10–12^. Although difficult to predict, climate change could lead to range shifts or constrictions^13^ and temporal separation of pollinators and the plants they pollinate. Herbicides are used regularly to control weeds in most cropping systems, and this may reduce the availability of season-long flowers for pollinators as well^14^.

Recognizing the importance of all pollinators and the threat of reduced floral resource and habitat, the U.S. National Strategy to promote the health of honey bees and other pollinators identified restoration of pollinator habitat acreage as one of its three main goals, aiming to restore or enhance 7 million acres of land for pollinators by 2020^15^. Pollination (and subsequently crop yields and quality) is generally higher in areas located closer to natural or semi-natural habitats^16,17^. Because of this, many researchers and policy makers are encouraging the creation of flower-rich habitats such as hedgerows, field borders, or cover crops to conserve bee populations and increase crop pollination^18,19^. However, it is still remain unclear whether these habitats actually increase the number of pollinators required for targeted crop pollination^20^, and if flowers in these habitats may compete with the crop and interfere with crop pollination^21,22^.

In recent past, impact of non-crop floral resources on different insect communities (such as pest and beneficial species or natural enemies of arthropod pests) in apple orchards has been studied^23–27^. However, season-long benefits to wild bee communities by establishing native perennial floral plantings in commercial apple orchards have not. Apple is a mass-blooming pollinator-dependent crop, and pollinator species visiting apple flowers during bloom also need floral resources after the short 7–10 days apple bloom period. Establishing floral plantings with a native perennial plant species mix with varying bloom times could provide season-long floral food resources to wild bee species in apple orchards and other agroecosystems. In the present study, the importance of establishing perennial floral plantings near commercial apple orchards was examined in Pennsylvania where a complex landscape with high plant diversity already exists^28^ and managed honey bees largely are not used commercially by the majority of growers^29^. In particular, we assessed the bee communities found in managed floral resource pollinator plantings and compared them to the bee communities found in nearby commercial apple orchards. In addition to our assessment of the entire bee community, we also examined how two subsets of the bee community – known tree fruit pollinators and rare species – compared between apple orchards and floral plantings. These subsets of the bee communities were selected to act as indicators of the utility of the floral resource plantings: known tree fruit pollinators represent value in improving crop pollination, and the presence of rare species indicates contributions to biological conservation in agricultural landscapes.

We expected that the bee communities in the orchard would be less rich and diverse than those in the floral plantings, because the floral plantings were designed to have diverse floral resources for a longer blooming period. However, we hypothesized that the orchards would have a more diverse and rich assemblage of bees that are known to be tree fruit pollinators because they would be spring-active species that are more likely to move into the orchard during fruit bloom periods. We also hypothesized that the floral plantings would support a higher abundance and richness of bee species identified as rare due to greater floral and nesting resources as compared to the apple orchards.

## Results

### Abundance

**Figure 1:**
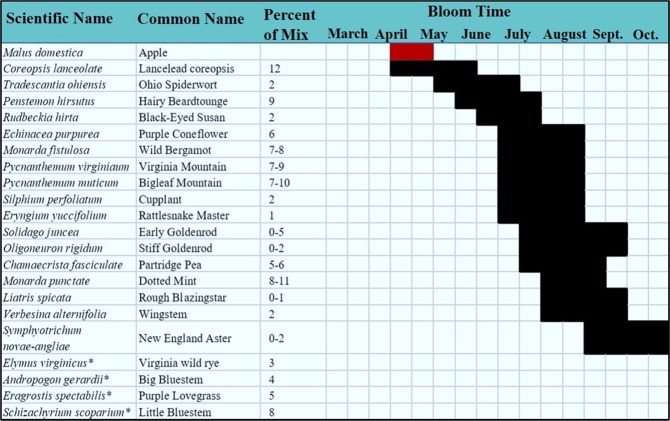
Plant species mix used for establishing floral plantings with their bloom times. *represents species of grasses in the seed-mix, and have no designated bloom time.

**Figure 2:**
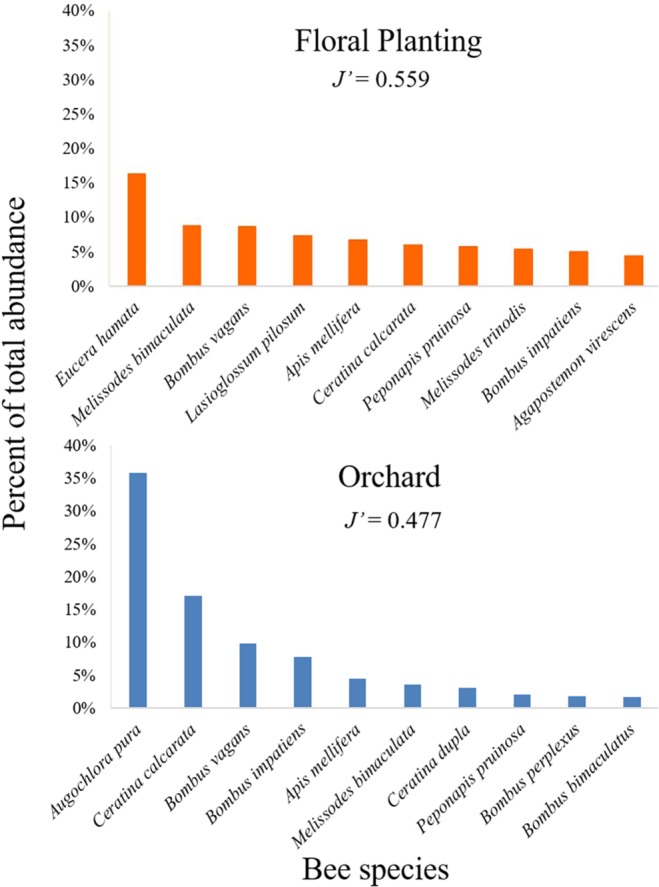
Percent abundance of the ten most abundant species in floral plantings and apple orchards. Pielou’s Index of Eveness (*J’*) values closer to 1 indicate more even distribution of abundance.

Over the three years of the study in multiple sites (Table 1), 24,155 bees were collected, 7,507 (156.4 bees per trap x site^−1^ x year^−1^) in the floral plantings and 16,648 (115.6 bees per trap x site^−1^ x year^−1^) from the apple orchards. Percent abundance varied greatly among species (Table 2). Out of the ten most common species in floral plantings established with different plant species mix (Fig. 1) and apple orchards, six species were found in both floral plantings and apple orchards (Fig. 2). However, the single most abundant species for each site type – *Eucera hamata* (floral plantings) and *Augchlora pura* (apple orchards) – were not among the most abundant in the other site type (Fig. 2). Based on Pielou’s evenness index, community evenness was higher in the floral plantings (*J’* = 0.559) than in the apple orchards (*J’* = 0.477). Across all samples collected, the known tree fruit pollinators were significantly more abundant in the orchards compared to the floral plantings (Z = 3.479, P = 0.0005). However, after apple bloom the abundance of known tree fruit pollinating bees in the floral plantings increased significantly (Z = −6.436, P = 1.23e^−10.^). Overall, there was no significant difference in rare species abundance between the orchards and the floral plantings (Z = − 0.137, P = 0.891).

**Table 1:** Details of apple orchard and floral planting sites.

Study Site	Size (ha)	Location
**Apple Orchards**		
KFL Round Barn	6.07	39°53′59.7′′N 77°21′08.2′′W
DS Piney Apple	4.05	40°00′25.9′′N 77°16′38.6′′W
DS RAMP	4.05	39°58′33.7′′N 77°19′10.6′′W
DS West	3.24	39°58′31.0′′N 77°19′47.9′′W
ED RAMP	3.64	39°56′35.7′′N 77°17′51.0′′W
SS HC	4.05	39°57′27.0′′N 77°17′16.3′′W
**Floral Plantings**		
BR Orchards	0.375	39°57′04.4′′N 77°14′54.7′′W
FREC-P	0.368	39°56′31.6′′N 77°15′39.0′′W
FREC-RLP	0.391	39°56′22.6′′N 77°15′01.9′′W
FREC-RLP-2	0.587	39°56′09.8′′N 77°14′55.9′′W
FREC-RSP	0.609	39°56′10.2′′N 77°14′56.9′′W
P-Cherryvale	0.616	39°57′31.0′′N 77°15′40.3′′W
SS North	0.258	39°57′19.0′′N 77°16′52.1′′W
SS South	0.174	39°57′04.9′′N 77°17′13.1′′W

**Table 2:** Species list and percent  abundances of bees in floral plantings and orchards in Adams Co., PA; 2012–2014.  = Known tree fruit pollinator, ◊ = Rare species

Species name	Author	Percent Abundance	
		Orchard	Floral Planting
**Andrenidae**			
*Andrena barbara*	Bouseman & LeBerge	0.01	0.01
*Andrena bisalicis*	Viereck	0.04	0.03
*Andrena carlini*	Cockerell	0.23	0.07
*Andrena commode*	Smith	0.26	0.13
*Andrena cornelli*	Viereck	0.01	
*Andrena cressonii*	Robertson		0.01
*Andrena distans* ◊	Provancher	0.01	
*Andrena dunningi*	Cockerell	0.50	0.03
*Andrena erythrogaster*	(Ashmead)	0.01	
*Andrena forbesii*	Robertson	0.02	0.04
*Andrena heraclei*	Robertson	0.02	
*Andrena hippotes*	Robertson	0.01	
*Andrena imitatrix*	Cresson	0.05	0.11
*Andrena mandibularis*	Robertson		0.01
*Andrena miserabilis*	Cresson	0.04	0.24
*Andrena nasonii*	Robertson	0.01	0.01
*Andrena nivalis*	Smith	0.01	
*Andrena nuda*	Robertson	0.01	
*Andrena perplexa*	Smith	0.41	0.12
*Andrena pruni*	Robertson	0.05	
*Andrena rugosa*	Robertson	0.05	0.01
*Andrena tridens*	Robertson	0.10	
*Andrena vicina*	Smith	0.02	
*Andrena violae*	Robertson	0.19	0.08
*Andrena wilkella*	(Kirby)	0.11	0.13
*Calliopsis andreniformis*	Smith	0.02	0.16
*Pseudopanurgus compositarum*	(Robertson)	0.01	
**Apidae**			
*Anthophora abrupta*	Say	0.16	0.01
*Anthophora bomboides*	Kirby	0.25	0.31
*Anthophora plumipes* ◊	(Pallas)	0.04	
*Anthophora terminalis*	Cresson	0.47	0.13
*Apis mellifera*	Linnaeus	4.41	6.61
*Bombus auricomus*	Robertson	0.01	0.07
*Bombus bimaculatus*	Cresson	1.71	2.42
*Bombus fervidus*	(Fabricius)	1.14	3.84
*Bombus griseocollis*	(DeGeer)	0.28	1.27
*Bombus impatiens*	Cresson	7.80	4.92
*Bombus insularis*	(Smith)		0.01
*Bombus perplexus*	Cresson	1.81	2.24
*Bombus vagans*	Smith	9.77	8.58
*Cemolobus ipomoeae* ◊	(Robertson)	0.02	0.08
*Ceratina calcarata*	Robertson	17.09	5.90
*Ceratina dupla*	Say	3.12	0.35
*Ceratina mikmaqi*	Rehan & Sheffield	0.01	
*Ceratina strenua*	Smith	0.35	0.16
*Eucera atriventris*	(Smith)	0.01	0.01
*Eucera dubitata*	(Cresson)	0.03	0.01
*Eucera hamata*	(Bradley)	1.21	16.21
*Eucera rosae* ◊	(Robertson)	0.01	
*Melissodes bimaculata*	(Lepeletier)	3.56	8.70
*Melissodes denticulata*	Smith	0.10	0.35
*Melissodes dentiventris*	Smith	0.01	0.08
*Melissodes desponsa*	Smith	0.86	0.97
*Melissodes druriella*	(Kirby)	0.02	0.04
*Melissodes illata*	Lovell & Cockerell	0.01	
*Melissodes subillata*	LeBerge		0.03
*Melissodes trinodis*	Robertson	0.08	5.33
*Melitoma taurea*	(Say)	0.45	0.60
*Nomada maculata*	Cresson	0.01	
*Nomada ovata*	(Robertson)	0.01	
*Peponapis pruinosa*	(Say)	2.09	5.66
*Ptilothrix bombiformis*	(Cresson)	0.17	1.11
*Svastra obliqua*	(Say)		0.04
*Triepeolus concavus* ◊	(Cresson)		0.01
*Triepeolus lunatus*	(Say)		0.01
*Xylocopa virginica*	(L.)	0.07	0.44
**Colletidae**			
*Colletes compactus*	Cresson		0.01
*Hylaeus affinis*	(Smith)	0.02	0.07
*Hylaeus hyalinatus*	Smith		0.03
*Hylaeus mesillae*	(Cockerell)		0.03
*Hylaeus modestus*	Say	0.07	0.01
**Halictidae**			
*Agapostemon sericeus*	(Forster)		0.05
*Agapostemon splendens*	(Lepeletier)		0.01
*Agapostemon texanus*	Cresson	0.05	0.29
*Agapostemon virescens*	(F.)	0.43	4.41
*Augochlora pura*	(Say)	35.74	3.44
*Augochlorella aurata*	(Smith)	0.91	1.29
*Augochlorella persimilis*	(Viereck)		0.01
*Augochloropsis metallica*	(Fabricius)	0.01	
*Halictus confusus*	Smith	0.05	0.11
*Halictus ligatus*	Say	0.37	3.61
*Halictus rubicundus*	(Christ)	0.15	0.03
*Lasioglossum admirandum*	(Sandhouse)	0.10	0.08
*Lasioglossum bruneri*	(Crawford)	0.02	0.01
*Lasioglossum callidum*	(Sandhouse)	0.01	0.07
*Lasioglossum coeruleum*	(Robertson)	0.01	
*Lasioglossum coriaceum*	(Smith)	0.07	0.03
*Lasioglossum cressonii*	(Robertson)	0.03	0.03
*Lasioglossum ephialtum*	Gibbs	0.01	0.01
*Lasioglossum forbesii*	(Robertson)	0.02	
*Lasioglossum foxii*	(Robertson)	0.04	
*Lasioglossum gotham*	Gibbs	0.04	0.01
*Lasioglossum hitchensi*	Gibbs	0.05	0.21
*Lasioglossum imitatum*	(Smith)	0.01	0.01
*Lasioglossum katherineae*	Gibbs	0.01	
*Lasioglossum macoupinense*	(Robertson)	0.01	
*Lasioglossum oblongum*	(Lovell)	0.01	0.01
*Lasioglossum obscurum*	(Robertson)	0.01	
*Lasioglossum paradmirandum*	(Knerer & Atwood)		0.01
*Lasioglossum pectorale*	(Smith)	0.01	0.03
*Lasioglossum pilosum*	(Smith)	0.43	7.23
*Lasioglossum quebecense*	(Crawford)	0.04	
*Lasioglossum subviridatum*	(Cockerell)	0.02	
*Lasioglossum tegulare*	(Robertson)	0.17	0.11
*Lasioglossum truncatum*	(Robertson)	0.02	
*Lasioglossum versans*	(Lovell)	0.05	0.04
*Lasioglossum versatum*	(Robertson)	0.14	0.04
*Lasioglossum weemsi*	(Mitchell)	0.01	
*Lasioglossum zephyrum*	(Smith)	0.01	
*Lasioglossum zonulum* ◊	(Smith)	0.01	
**Megachilidae**			
*Anthidium manicatum*	(Linnaeus)		0.12
*Anthidium oblongatum*	(Illiger)	0.01	0.09
*Coelioxys rufitarsis*	Smith	0.01	
*Coelioxys sayi*	Robertson	0.01	
*Heriades carinata*	Cresson		0.04
*Hoplitis pilosifrons*	(Cresson)	0.01	0.04
*Hoplitis truncate*	(Cresson)		0.01
*Megachile addenda*	Cresson		0.01
*Megachile brevis*	Say	0.02	0.07
*Megachile campanulae*	(Robertson)	0.02	0.09
*Megachile centuncularis*	(Linnaeus)		0.01
*Megachile gemula*	Cresson	0.05	
*Megachile integra*	Cresson	0.01	
*Megachile melanophaea*	Smith	0.01	
*Megachile mendica*	Cresson	0.08	0.08
*Megachile montivaga*	Cresson		0.05
*Megachile pugnata*	Say	0.02	
*Megachile relativa*	Cresson	0.01	
*Megachile rotundata*	(Fabricius)		0.08
*Megachile sculpturalis*	Smith		0.03
*Osmia atriventris*	Cresson	0.31	0.05
*Osmia bucephala*	Cresson	0.16	0.08
*Osmia cornifrons*	(Radosz.)	0.20	0.01
*Osmia lignaria*	Say	0.03	0.03
*Osmia pumila*	Cresson	0.50	0.03
*Osmia taurus*	Smith	0.05	0.01
*Osmia texana* ◊	Cresson	0.01	0.01
*Osmia virga*	Sandhouse	0.01

### Richness

**Figure 3:**
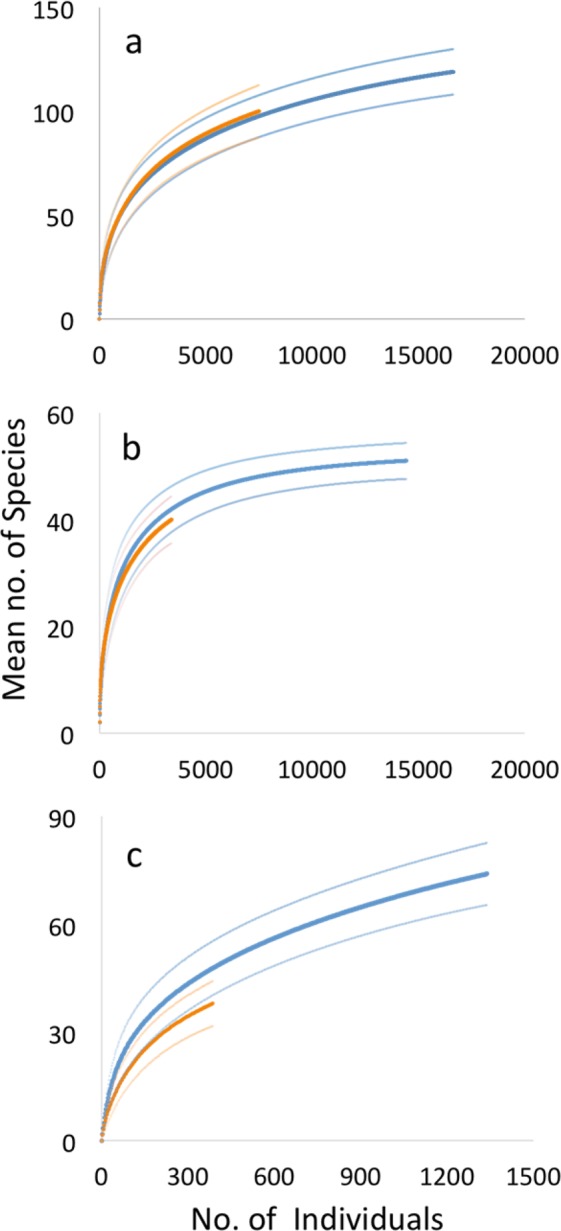
Individual-based rarefaction curves with 95% confidence intervals depicting (**a**) full season samples, (**b**) known tree fruit pollinator species, and (**c**) bloom time species accumulation in orchards (blue) and floral planting (orange).

Over the course of the study, 138 bee species were identified, of which 100 species were recorded in the floral plantings and 116 species in the orchards (Table 2). Fifty-eight species were identified as tree fruit pollinators and six species were categorized as rare (Table 2). There was no difference in bee species richness between floral plantings and apple orchards for all species collected (Fig. 3a) and for known tree fruit pollinating species (Fig. 3b), as species accumulation occurred at almost identical rates in both site types. A higher rate of species accumulation was observed in the apple orchards as compared to floral plantings during the apple bloom time (Fig. 3c), although 95% confidence intervals still overlapped.

### Diversity

**Figure 4:**
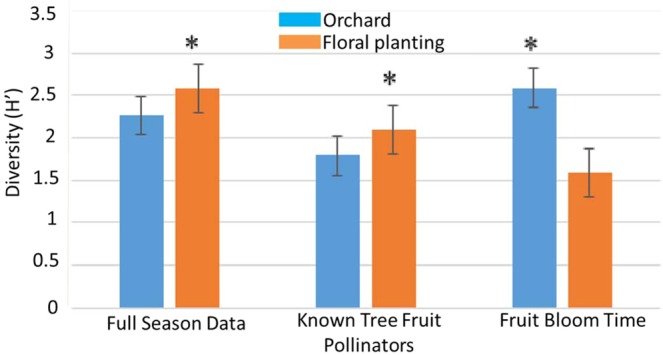
Mean Shannon Diversity Index for orchard sites versus floral plantings for the full season (full data set), for the known tree fruit pollinator species and for samples collected during fruit bloom. Error bars represent standard error.

Over the course of the entire study, bee communities in floral plantings were more diverse than those in the orchards based on Shannon index values (Z = −3.219, P = 0.0013) (Fig. 4). The floral plantings also had a more diverse community of known tree fruit pollinators than the orchards (Z = −2.056, P = 0.0397) (Fig. 4). However, during bloom time only the bee communities in apple orchards were more diverse than in floral plantings (Z = 8.083, P = 6.66e-16) (Fig. 4).

### Community assemblages

**Figure 5:**
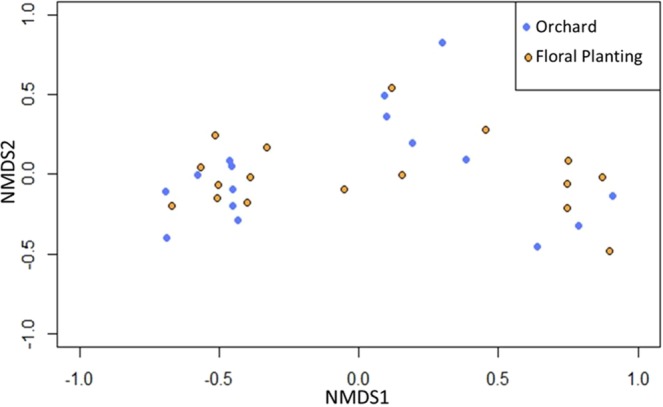
Non-metric multidimensional scaling ordination of study sites and types according to bee species composition. The ordination is based on the relative Sørensen index, which separates sites based on proportional abundance (Stress = 0.1136, P = 0.816).

**Figure 6:**
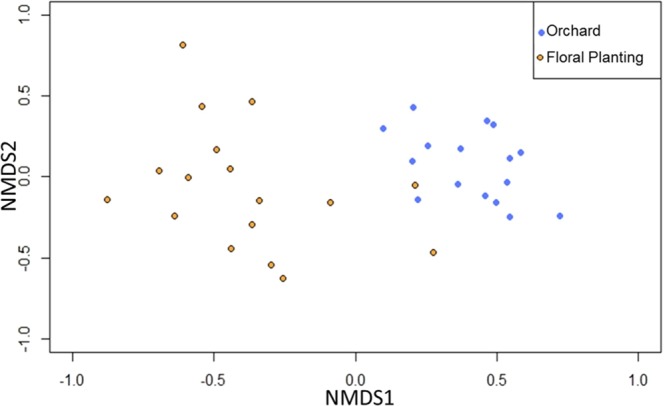
Non-metric multidimensional scaling ordination of study sites and types according to known tree fruit pollinating bee species composition. The ordination is based on the relative Sørensen index, which separates sites based on proportional abundance (Stress = 0.1395, P = 0.001).

**Figure 7:**
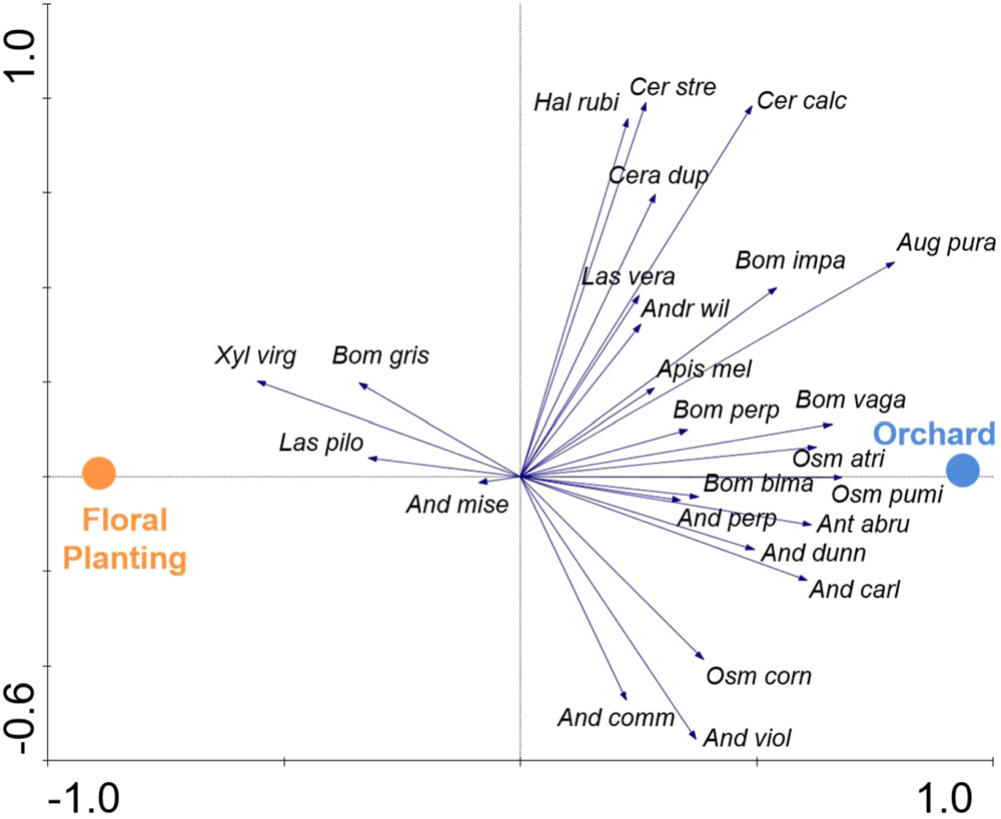
RDA ordination biplot depicting associations between site types and known tree fruit pollinating species. Species names have been abbreviated; full names are in Table 2.

The bee communities visiting orchards and floral plantings were not differentiable in the non-metric multidimensional scaling (NMDS) ordination (Stress = 0.1136, P = 0.816) (Fig. 5). However, when the data were limited to known tree fruit pollinators, the communities visiting the orchards and the floral plantings were significantly different (Stress = 0.1395, P = 0.001) (Fig. 6). Among these known tree fruit pollinators, there were distinct community assemblages (F = 8.51, P = 0.002) between orchards and floral plantings, with most species being more closely associated with the orchards (Fig. 7). The first axis of the RDA biplot, which delineated the community gradient between the two site types, explained 22.7% of the variance in species data.

## Discussion

Assuring sufficient pollination services is a critical component of commercial apple production. Due to the increasing rental costs of honey bees, growers may rely more on pollination services from wild, non-*Apis*, bees^9,29^. In addition, in certain cropping systems and landscapes, wild bees have been shown to visit crops at higher rates than honey bees^27,30,31^. Growers relying on pollination services from wild bees would thus benefit from adopting practices that actively promote abundant and diverse bee communities as increased pollinator density and diversity has been linked to improve crop yields^32–34^. Our study examined the contribution of season-long floral resource plantings to the wild bee communities in apple orchard agro-ecosystems, and to our knowledge, it is the first multi-year study documenting such season-long utility of perennial floral plantings in commercial apple orchards.

Over the course of the study, we found a diverse array of bees in both the floral plantings and the orchards. An analysis of species assemblages found that the community composition was not different between the habitat types, when the bee community was sampled over the entire season. Similarly, species richness, based on individual-based rarefaction analysis, was also not different between the orchards and floral plantings when compared seasonally. However, diversity indices suggested that the overall diversity and evenness of the bee community (based on Shannon Index and Pielou’s Index, respectively) were greater in the floral plantings. This was likely due the increased plant diversity and availability of season-long floral resources – traits that are often associated with increased pollinator diversity^35–37^. Although the pre-existing orchard/forest interface habitat in Pennsylvania has been found to support a large population of plant species^28^, the floral plantings used in this study were designed to bloom for a much longer period of time than the orchard/forest interface (see Fig. 1). Many of the plants in the orchard/forest interface in this region are early season perennials^28^. It is also possible that the orchard/forest interface is impacted by orchard pesticide application drift that could reduce both plant diversity and bee diversity.

Since the seed mixes of the floral plantings were constructed to minimize bloom phenology overlap with apples (Fig. 1), we conducted additional analyses to see if the bee communities responded to these temporal and spatial shifts in floral resources as documented in other settings, such as landscapes with co-blooming crops^38^. When analyses were restricted to pollinators collected during the tree fruit bloom period only, apple orchards supported a greater diversity of bees and tended to have higher species richness levels. The species assemblage patterns differed significantly between orchards and floral plantings during bloom period, as well. In addition, most of the bees characterized as known apple pollinators (based on previous net collections from flowers), also showed a close association with orchards in this study (see Fig. 7). Of the species most highly associated with apples, three species – *Augochlora pura*, *Ceratina calcarata*, and *Bombus vagans* – comprised more than half of the total bee abundance found in orchards. These species are all known tree fruit pollinators that are commonly found in the orchards in this area^39–41^. *A*. *pura* and *C*. *calcarata* nest in rotting wood, stems, or pith, which makes the orchard-forest interface an ideal habitat for them and allows these species to thrive in this landscape. In addition, some of the other species associated with the orchard, such as *Osmia* spp. and *Andrena* spp., are early season univoltine species, whose foraging activities coincide with apple bloom, thus making them less reliant on the later-blooming flowers in the pollinator plantings. Among the list of known tree-fruit pollinators, only *Xylocopa virginica*, *Bombus griseocollis*, and *Lasioglossum pilosum* were noticeably associated with the floral plantings. All three of these species are generalists, which may be utilizing the late season blooming resources in the floral plantings.

The different diversity and assemblage patterns between whole season and apple bloom period analyses suggests that floral plantings, if properly designed, may serve as an important reservoir for some known tree fruit pollinators when the crop is not in bloom and can contribute to overall pollinator conservation in agricultural landscapes. The diverse plant community in the floral plantings attracted a diverse set of tree fruit pollinating species. Such diversified floral plantings may be acting as a nest site resource, as well as a reservoir for tree fruit pollinators, as we recorded an increase in the abundance of tree fruit pollinating species in the pollinator plantings after fruit bloom. In addition, as introduced landscape elements, the floral plantings seem to work in a complementary fashion with apple orchards in terms of promoting pollination services: known tree fruit pollinators readily moved into the apple orchards during bloom and the floral plantings did not seem to act as a pollinator ‘sink’ during this time period. There was no difference in the abundance of rare species in the orchards and the floral plantings, although detecting differences would likely always be difficult due to the inherently low abundance of these species. *Triepeolus concavus* was the only rare species found in the pollinator plantings and not the orchards, and is a known nest parasite of the bee genus *Colletes*.

The floral plantings met the design goals of providing season-long support to pollinator communities in apple orchards in this region. Anecdotally, we did not observe plant species in the pollinator plantings becoming weeds in the orchards, or an increase in pests that used the pollinator planting species as hosts. Furthermore, when the data were limited to only bees collected during fruit bloom, the orchards were significantly more diverse than the floral plantings, indicating that these plantings were not attracting pollinators away from the orchards during fruit bloom. However, we did witness problems with weed management and sustainability of the floral plantings. Canadian thistle (*Cirsium arvense*), poison ivy (*Toxicodendron radicans*), Virginia creeper (*Parthenocissus quinquefolia*) and field bindweed (*Convolvulus arvensis*) were abundant in many of the plantings and required spot treatment with herbicides.

Overall, the floral plantings attracted a more diverse community of pollinators, and in order to act optimally as a pollination service supplementation, they must be tailored more specifically to the crop and the species that pollinate it. Only a small minority of common bee species provide most of the crop pollination services^42^, so those bees must be targeted when creating pollinator habitat. For example, it would have been beneficial to have more early season plants in floral plantings, blooming before the crop in the pollinator habitats, which may better support apple pollinating species most of which emerge several weeks before apple bloom^27^, which include early season bees such as *Andrena* spp. and *Osmia* spp.^43^. These floral plantings increase populations of wild bees and other flower visiting insects in apple orchards^27^, but other strategies such as developing management practices for hedgerows and woods-edge^43^ could also benefit pollination supplementation and bee conservation in commercial apple orchards.

## Materials and Methods

### Study sites

Multi-year field studies were conducted at six separate apple orchards and eight floral plantings (often referred to as pollinator strips) on commercial tree fruit farms in Adams County, PA from 2012–2014 (Table 1). In this region of Pennsylvania, which is the leading tree fruit producing county in the state, orchards have steep slopes, well drained soils and a landscape matrix of approximately 8% orchards, 24% arable and pasture land, 9% developed area and 56% forests^44^. Orchards are generally small and bordered by undeveloped scrub, forest, or fence rows. This orchard-forest interface is home to a diverse plant community, supporting at least 146 plant species^28^ and 188 species of bees from a collection of 80,000 field collected bees (DJB unpublished data). Because of the high bee diversity in this complex landscape, and a tripling in the cost of honey bee colony rentals since 2006, the majority of growers in this region do not rent honey bee colonies for pollination and rely completely on wild bees for pollination^29^. The study orchards ranged in age from 15–25 years, and contained primarily ‘York’, ‘Golden Delicious’, and ‘Honey Crisp’ apple varieties, and none of these orchards used managed honey bees for pollination, nor were within 1 km of managed honey bee hives.

Floral plantings were established under a project funded through contracts between Pennsylvania fruit growers and the United States Department of Agriculture’s Natural Resource Conservation Service (USDA-NRCS) under the Environmental Quality Incentive Program (EQIP) and Conservation Stewardship Program (CSP). These floral plantings were established according to guidelines previously developed cooperatively by the Xerces Conservation Society, Penn State University and Pennsylvania USDA-NRCS. The guidelines specified seeding rates, plant species, and establishment and maintenance programs^45^. All floral plantings (except one) were established in 2011 and 2012. Site location and other related information of these plantings are given in Table 1.

The seed mixtures for floral plantings were supplied by Ernst Conservation Seed (884 Mercer Pike, Meadville, PA 16335) to best support a diversity of local bees throughout the season (see Fig. 1). Working cooperatively with USDA-NRCS and the fruit growers we established all floral plantings next to apple orchards, with a focus on: (a) providing blooming wild plants throughout the growing season, while minimizing overlap with apple bloom time; (b) including plant species that would not become weeds in apple or other fruit crops; and (c) including plant species that would not serve as alternate hosts of crop diseases or pests, such as brown marmorated stink bug. Orchards were maintained similarly by conventional IPM practices, which included both conventional and reduced risk pesticides as well as biological control. Floral plantings were maintained with yearly mowing and spot spraying for noxious weeds with selective herbicides, in accordance with the USDA-NRCS/Xerces Society recommendations.

### Bee sampling in floral plantings and apple orchards

We sampled bee populations in the orchards and the floral plantings using blue vane traps (model Z-BVT, SpringStar Inc., Woodinville, WA), which are highly effective in sampling pollinators^46,47^. The blue vane trap (BVT) consists of a fluorescent yellow plastic container (0.71 L volume capacity) with a blue plastic funnel and two blue plastic cross vanes. Each floral planting was monitored with two BVT traps, while the larger orchard blocks were monitored with five or ten traps. Traps were suspended approximately 1.5 m above the ground with nylon rope from a metal pole. Supertech® antifreeze (a mixture of ethylene glycol and diethylene glycol, Wal-Mart Stores, Inc., Bentonville, AR) diluted with tap water (60:40) was used as drowning media and specimen preservative for these traps. Trapped bees were collected weekly, from early April to first frost (~mid-October) each year.

Bees were collected from traps using a stainless steel strainer and a pair of flathead forceps, and immediately placed in a clear plastic vial containing 70% ethanol for further processing. In the laboratory, bee samples were dried, pinned, labeled, and identified to species level. Identifications were done by J. Gibbs at Michigan State University (*Lasioglossum* spp.), R. Jean at Saint Mary-of-the Woods College (Andrenidae samples), and K. Wright at the University of New Mexico (*Melissodes* spp.). The remaining bees were identified by R. Donoval (USDA-APHIS) and D. Biddinger (Penn State University) using keys^48–50^, and Discover Life ID guides (www.discoverlife.org).

### Statistical analysis

Prior to analysis, data were combined across dates and traps at each site within a year. In addition, some analyses were performed on subsets of the bee community, specifically: known tree fruit pollinators (determined by previous net collections in Pennsylvania orchards at pome and stone fruit bloom), rare species for Pennsylvania (determined by expert opinion, S. Droege and D. Biddinger), and only bees collected in BVT during tree fruit bloom time (mid-April to mid-May).

#### Abundance

Rank abundance curves for the ten most abundant species in each habitat were plotted to identify the most common species found in floral plantings and apple orchards, respectively. Community evenness overall was compared among habitats using Pielou’s index of evenness (*J’*)^51^. Percent abundance of species identified as known fruit pollinators and species identified as rare was calculated for each site in each year, and analyzed in RStudio using generalized linear mixed models using the lme4 package^52,53^, in which site and year were assigned as random factors, and site type (orchard versus floral planting) as a fixed explanatory factor.

#### Richness

Since the number of samples differed between floral plantings and orchards, species richness was compared between site types using individual-based rarefaction curves developed in EstimateS 8.2^54^. The rarefaction curves allow for a comparison of species richness by indicating the statistical expectation of species accumulation based on 100 permutations of the species-by-sample matrix. Differences in species accumulation between apple orchards and floral plantings were determined by non-overlapping confidence intervals^55^.

#### Diversity

The Shannon’s diversity index (H’), which accounts for both species richness and evenness, was calculated for each site-year combination. Diversity index means were compared in RStudio using generalized linear mixed models using the lme4 package^52,53^ in which site and year were assigned as random factors, and site type (orchard versus floral planting) as a fixed explanatory factor.

#### Species assemblages

Differences in species composition of the bee communities in the orchards versus those in the floral plantings were visualized with non-metric multidimensional scaling (NMDS) using the metaMDS function in the vegan package in RStudio^52,56^. NMDS is an ordination technique used to visualize the similarity between data points (in this case, each site/year combination) based on the dissimilarity of any number of variables (in this case, the abundance of each species)^57^. We conducted two NMDS analyses; the first analysis included all bee species collected in this study, and the second analysis only included the subset of bees that were known to be tree fruit pollinators. Statistical significance was determined by a PERMANOVA using the adonis function in the vegan package in RStudio^52,56^.

Associations in community assemblage patterns between individual tree fruit pollinating species and site types (orchard and floral plantings) were further examined using redundancy analysis (RDA), a constrained ordination technique, in CANOCO 4.5^58^. Species data were centered and standardized, and year was included as a covariate in the analysis. The significance of site type was determined using Monte Carlo simulations with 499 permutations of the data. Species associations with orchard or floral planting were then visualized using biplots developed in CanoDraw 4.5^58^.
