# Publisher Correction: Diversified Floral Resource Plantings Support Bee Communities after Apple Bloom in Commercial Orchards

**DOI:** 10.1038/s41598-020-58960-1

**Published:** 2020-02-21

**Authors:** Sarah Heller, Neelendra K. Joshi, Timothy Leslie, Edwin G. Rajotte, David J. Biddinger

**Affiliations:** 1 Fruit Research & Extension Center, Entomology, Pennsylvania State University, 290 University Dr, Biglerville, 17307 PA USA; 2 Department of Entomology, 501 ASI Building, Pennsylvania State University, University Park, 16802 PA USA; 3 Present Address: Department of Entomology and Plant Pathology, 217 Plant Sciences Building, University of Arkansas, Fayetteville, Arkansas 72701 USA; 4 Department of Biology, Long Island University, 1 University Plaza, Brooklyn, New York 11201 USA; 5 Present Address: USDA APHIS PPQ Otis Laboratory, 1398 West Truck Road, Buzzards Bay, Massachusetts 02542 USA

Correction to: *Scientific Reports* 10.1038/s41598-019-52601-y, published online 21 November 2019

The original HTML version of this Article contained errors in Table 2, where the incorrect symbol was used to represent known tree fruit pollinators. This has now been corrected in the HTML; the PDF version of the paper was correct from the time of publication.

